# Decoupling Between Functional Diversity and Stability of Decomposer Functions in Natural and Agroecosystems Can Favor Resistance to Land‐Use Change

**DOI:** 10.1002/ece3.72190

**Published:** 2025-09-20

**Authors:** Shamik Roy, Sumanta Bagchi

**Affiliations:** ^1^ Centre for Ecological Sciences Indian Institute of Science Bangalore India; ^2^ Forest Zoology Technische Universität Dresden Tharandt Germany

## Abstract

Functional diversity in producers and consumers can not only promote ecosystem functions but also impart additional desirable features, such as stability of functions and how they are translated into services. These connections between diversity, functions, and stability are seen across various natural ecosystems in the terrestrial, marine, and freshwater realms. Yet, it remains challenging to extend these linkages to agroecosystems—because producers and consumers play widely different ecological roles here compared to natural ecosystems. Microbial decomposer functions, however, are common to both natural and agroecosystems. But the linkages between functional diversity in decomposers, their functions, and stability remain inadequately known. We take advantage of human land‐use in the Trans‐Himalayas where the natural reference grazing ecosystem with native plants and herbivores is repurposed into two distinct agroecosystems to grow livestock or crops. Here, we answer three questions: (a) whether land‐use change alters the intensity of decomposer functions, (b) whether land‐use change homogenizes decomposer functions, and (c) whether land‐use change alters the stability of decomposer functions. Variation in decomposer functions was not attributable to background spatial autocorrelation or variation in edaphic conditions. We find that the intensity of the individual decomposer functions was higher under crops compared to the native state, but the intensity remained comparable under livestock. Land‐use had no net effect on multifunctionality. The functional diversity was lower under crops and was comparable under livestock. We find that land‐use did not affect the temporal stability of the decomposer biomass. Structural equation models further suggested that functional diversity is decoupled from the stability of decomposer biomass. These results indicate that decomposer functions can be resistant to change in land‐use. Therefore, ecological resistance in decomposer functions can offer the basis for stewardship of agroecosystems since homogenization can result in ecosystems becoming more susceptible to environmental fluctuations, such as those foreseen by future climate projections.

## Graphical abstract

The links between microbial functional diversity and ecosystem stability remain unclear. Microbial functions vary more temporally than spatially, and ecosystem stability is decoupled from functional diversity in agroecosystems.

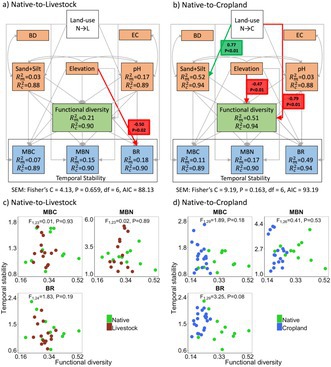

## 1 Introduction

Functional diversity, regardless of taxa or trophic position (Mason et al. 2005), is represented by the range of values of traits or functions of different organisms that can be a key determinant of ecosystem functions (Tilman 2001; Malaterre et al. 2019). Functionally diverse assemblages can be high functioning and also be able to maintain high levels of function through time (i.e., stability) (Tilman 2001; Díaz and Cabido 2001; Cadotte 2017). Repeated demonstration of these facets of functional diversity, across various taxa in terrestrial, freshwater, and marine settings (McGrady‐Steed et al. 1997; Naeem and Li 1997; Ives et al. 1999; Engelhardt and Ritchie 2001; Duffy 2002; Hooper et al. 2005; Cardinale et al. 2011), implies that threats to functional diversity can jeopardize reliable provisioning of ecosystem services (Hooper et al. 2005; Cadotte et al. 2011). Land‐use change from natural ecosystems to agroecosystems is a direct threat to functional diversity, which imperils essential ecosystem functions and services at ever‐increasing rates (Tilman et al. 1997; Steffen et al. 2007). However, compared to producers and consumers, less is known about the functional consequences of land‐use change for belowground microbial decomposers.

Microbial decomposers influence key ecosystem processes and ultimately regulate biogeochemical cycles and global climate that are common across natural and agroecosystems (Kennedy 1999; Falkowski et al. 2008). Land‐use change can fundamentally alter ecosystem functions via its effects on microbial functional diversity. Further, decomposer functions are performed by extracellular enzymes that break down organic matter (Zhou et al. 2020; Roy and Bagchi 2022). These enzymes are relatively long‐lived in soil, with turnover times that exceed the typical generation time of individual microbial cells (Allison 2006; Rousk and Bååth 2011). So, unlike producers and consumers, ecological functions can overlap with multiple generations of decomposer organisms (Allison 2006; Rousk and Bååth 2011). Thus, for decomposers, functional diversity is best assessed at the level of the whole assemblage rather than as individual contributions from participant species or taxa (Zhou et al. 2020; Roy, Karapurkar, et al. 2023). Here, measurements of enzymatic functions can be more informative than documenting the metagenomic potential for functions that are embedded within nucleic acids, since the presence of the genetic potential may not always translate into functional performance, that is, “potential” vs. “realized” functions (New and Brito 2020).

Land‐use change, where a native ecosystem is repurposed to grow crops or to raise livestock, offers an important opportunity to address the outstanding questions on functional diversity and stability of decomposers in natural and agroecosystems. Here, we study three inter‐related questions. First, we investigate whether there is a difference in the intensity of decomposer functions between natural and agroecosystems. We define the intensity of the decomposer functions as the total activity of the extracellular enzymes. Second, we investigate whether land‐use change from natural ecosystems to agroecosystems relates to the homogenization of decomposer functions. Third, since microbial functions show marked phenological dynamics in seasonal environments (Bagchi et al. 2017), we investigate whether the temporal stability of decomposer functions also varies between natural and agroecosystems across different seasons. The temporal scale in which the community structure of microbial decomposers changes ranges from days to months, and we address intra‐annual stability across the growth season (Lauber et al. 2013; Shade et al. 2013; Wagg et al. 2021). From these, we draw inferences on the link between functional diversity and stability for decomposers in agroecosystems relative to the reference natural ecosystem. The linkages between functional diversity and stability are evident for producers and consumers, where a highly functioning producer or consumer community is stable to seasonal variation, primarily because of functional redundancy (Tilman 2001; Ives and Carpenter 2007; Cadotte 2017).

To address these questions, we measured seven decomposer functions involved in the mineralization of soil organic matter across land‐use types at different points in the growth season (Table 1). Decomposer functions can show greater variation through time than across space (Bardgett et al. 2005; Frossard et al. 2012; Roy, Karapurkar, et al. 2023). This difference in spatial versus seasonal responsiveness gets support in the adage “everything is everywhere, but the environment selects” (Bass‐Becking 1934). From this, we generated alternative a priori hypotheses and expectations on the basis of the literature (Frossard et al. 2012; Olivier et al. 2020; Zhou et al. 2020; Roy, Naidu, et al. 2023; Roy, Karapurkar, et al. 2023):

**TABLE 1:** Summary description of different microbial traits and their ecological functions.

Microbial trait	Units	Function
Beta‐Glucosidase (BG)	nmol g^−1^ soil h^−1^	Decomposition of cellulose to release glucose—a major source of energy for microbes
Cellobiohydrolase (CBH)	nmol g^−1^ soil h^−1^	Hydrolysis of cellulose to yield cellobiose, a disaccharide
Leucine Aminopeptidase (LP)	nmol g^−1^ soil h^−1^	Degradation of peptides with leucine residues at the N‐terminal end, to yield free amino acids
Beta‐*N*‐acetylglucosaminidase (NAG)	nmol g^−1^ soil h^−1^	Hydrolysis of chitin which is a polymer of amino‐sugars, to yield N‐Acetylglucosamine
Phophatase (P)	nmol g^−1^ soil h^−1^	Releases phosphate from a variety of compounds for microbial uptake to build cellular structures and nucleic acids
Polyphenol Oxidase (PPO)	nmol g^−1^ soil h^−1^	A multi‐purpose enzyme that degrades phenolic compounds, regulates the redox state of soil, and participates in humification
Peroxidase (PO)	nmol g^−1^ soil h^−1^	Decomposition of the recalcitrant fraction of soil organic matter using H_2_O_2_ as the electron acceptor. Majorly secreted by fungi
Basal respiration (BR)	mg C g^−1^ soil day^−1^	Soil organic carbon mineralization for efflux of carbon into the atmosphere
Microbial biomass‐Carbon (MBC)	mg C g^−1^ soil	Immobilization of Carbon
Microbial biomass‐Nitrogen (MBN)	mg N g^−1^ soil	Immobilization of Nitrogen

H1—Decomposer functions are likely to vary more with time (intra‐annual timescale) than across space because of the inherent seasonal dynamics of nutrient input (Bardgett et al. 2005; Frossard et al. 2012; Roy, Naidu, et al. 2023; Roy, Karapurkar, et al. 2023), or

H1′—Alternatively, decomposer functions can vary more across space than with time when there is variability in abiotic and biotic properties between samples (De Deyn and Van Der Putten 2005; Fierer and Jackson 2006).

H2—Decomposer functions are likely to be lower in agroecosystems compared to the reference native state because of a lower aboveground biomass input in agroecosystems (Spehn et al. 2000; De Deyn and Van Der Putten 2005),

H2′—Alternatively, decomposer functions can be higher in agroecosystems compared to the reference native state because of stress and nutrient limitation for microbes in agroecosystems (Sinsabaugh and Moorhead 1994; Allison and Vitousek 2005; Allison 2005; Sinsabaugh et al. 2009; German, Chacon, and Allison 2011; Sinsabaugh and Follstad Shah 2012; Roy and Bagchi 2022),

H3—Decomposer functions will be less heterogeneous in agroecosystems compared to the reference native state due to lower spatial variability (Spehn et al. 2000; De Deyn and Van Der Putten 2005),

H3′—Alternatively, heterogeneity of decomposer functions can be resistant to land‐use change, and

H4—If the link between functional diversity and stability seen in producers and consumers is generalizable, then functional diversity in microbes will likely favor the stability of decomposer biomass through time,

H4′—Otherwise, functional diversity in microbes will not influence the stability of decomposer biomass through time.

## 2 Materials and Methods

### 2.1 Study Site and Sampling

**FIGURE 1:**
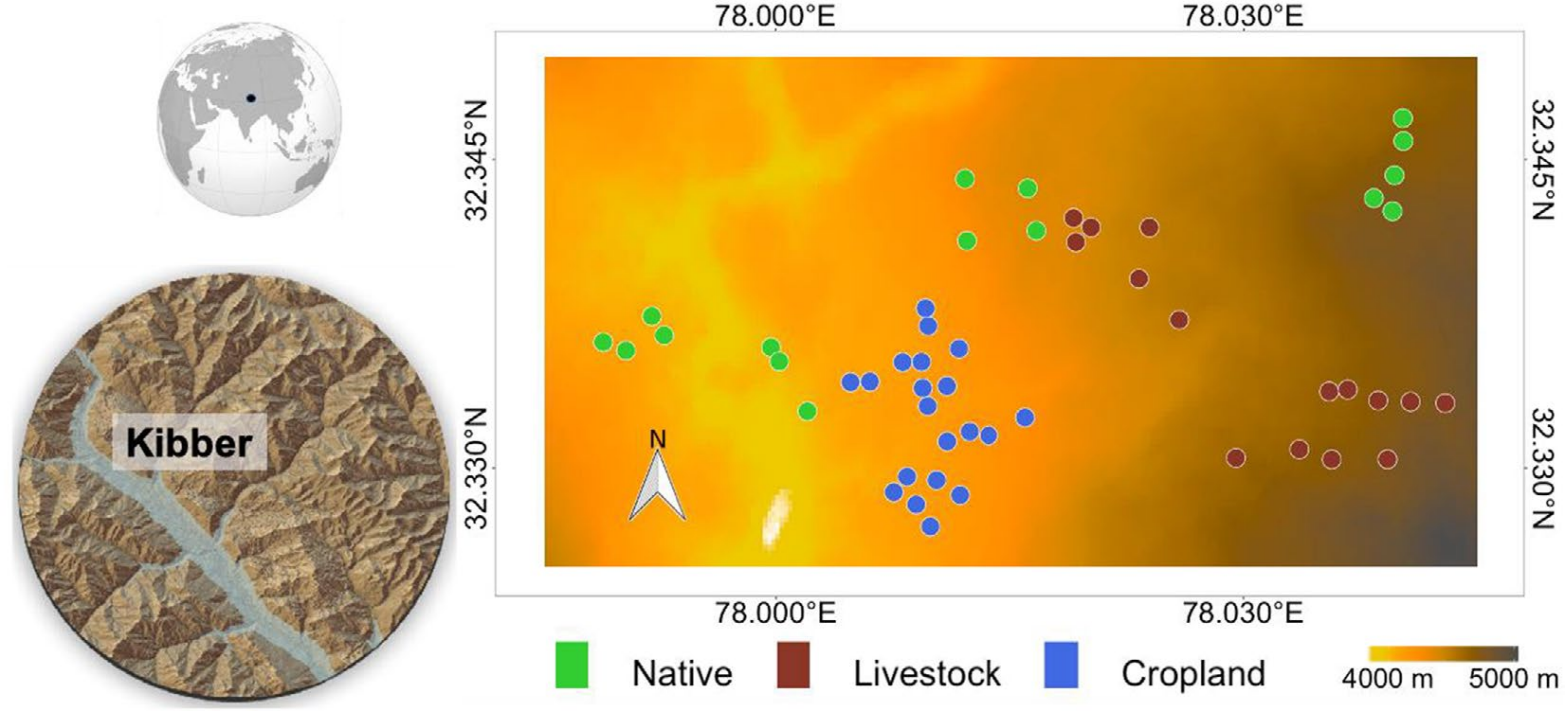
Map showing study area and sampling locations across three land‐use categories near village Kibber in the Spiti region of northern India. Sampling locations were in native reference state (*n* = 16), livestock (*n* = 15) and croplands (*n* = 20), distributed across an area of c. 26 km^2^. These locations were permanently marked for soil sampling in May, July, and September 2017.

We take advantage of the juxtaposition of land‐use as natural and agroecosystems at our study site near village Kibber (32° N, 78° E, 4200–4500 m asl) in the Spiti region of northern India (Figure 1). This provides a unique opportunity to study the functional consequences of change in the native shrub‐steppes to livestock, or to croplands, with replicates of each in distinct watersheds across the mountainous landscape. Spiti is a part of the Trans‐Himalayan eco‐region—a vast high‐altitude shrub‐steppe spread across the Central Asian highlands (Figure 1). The natural reference state is a shrub‐steppe grazed by native large mammalian herbivores (ibex, *Capra sibrica*; bharal, *Pseudois nayaur* ; yak, *Bos grunniens* ). The pastoral state arises when domestic livestock (goat, sheep, donkey, horse, cattle, and yak‐cattle hybrids) replace the native herbivores as consumers, but wild plants continue to be the producers. Next, the most simplified land‐use is croplands, where consumers are removed, and wild plants are replaced by monocultures of pea and barley.

Sampling for native, livestock, and cropland sites was distributed in 10 replicated watersheds that are separated from each other by natural terrain (high ridges, deep canyons, or streams and rivers, etc.). Four of these watersheds are primarily grazed by native herbivores, four are primarily grazed by livestock, and two contain croplands. We sampled 3–5 sites in each watershed under native herbivores and livestock, such that there were 16 native sites and 15 livestock sites. We sampled from 20 sites in the two watersheds under cropland, with one watershed consisting of six sites and the other 14 sites.

The climate in Spiti is cold and subhumid, and highly seasonal with a short growth season for plants between May and August (Bagchi et al. 2017). Ambient daytime temperatures drop below −30°C during the winters, reach 15°C in spring, peak during summer (25°C), and begin to drop again in autumn (15°C) (Figure S1). Precipitation occurs as snow (100–200 cm, November–March) and rain (150–300 mm, July–August) (Figure S1). Soils are near‐neutral to alkaline with coarse (sandy‐loam) texture (Figures S2 and S3) (Bagchi and Ritchie 2010; Bagchi et al. 2017). There is interannual variation in microbial functions, but given the temporal scale (10^−4^–10^−1^ years) at which microbes operate in soil, we address the phenological differences in them at an intra‐annual scale. We sampled in 2017, which was an average (typical) year in terms of temperature, precipitation, and vegetation growth (Figure S1).

### 2.2 Soil Enzymatic Activity as Decomposer Functions

In 2017, we sampled soil with a 2.5 cm diameter and a 10 cm deep corer three times in the growing season: May (early season), July (peak season), and September (senescence), in the three land‐use types from permanently marked sampling locations (Figure 1). The number of replicates was *n* = 16 for the native reference state, *n* = 15 for livestock, and *n* = 20 for croplands, for a total of 51 locations spread across an area of c. 26 km^2^ (Figure 1). Soils were transported to the laboratory, and we measured extracellular enzyme activities within 5 days of collection. We measured the activities of five hydrolytic and two oxidative enzymes to cover a wide range of decomposer functions involved in the recycling of soil organic matter (Table 1 and Table S1). Hydrolytic enzymes were beta‐glucosidase (BG), cellobiohydrolase (CBH), N‐acetylglucosaminidase (NAG), leucine aminopeptidase (LP), and phosphatase (P). Oxidative enzymes were peroxidase (PO), and phenol oxidase (PPO). We followed standard laboratory protocols (Saiya‐Cork et al. 2002; German, Weintraub, et al. 2011). Briefly, we prepared sample homogenates by adding 1 g of soil to 100 mL of 0.05 M Tris buffer of pH 7.8 and shaking the mixture for 1 min. We added fluorogenic or chromogenic substrates specific for each enzyme to the slurry (Table S1). We incubated for 2 h for hydrolytic enzymes and for 15 h for the oxidative enzymes. For the hydrolytic enzymes, we added 10 μL of 2 M NaOH at the end of the incubation period to stop the reaction. We took fluorogenic measurements in a fluorimeter (Tecan Infinite M200 Pro, Mannedorf, Switzerland) at an excitation wavelength of 365 nm and an emission wavelength of 450 nm. For colorimetric reactions, we measured spectral absorbance at a 460 nm wavelength in 100 μL of the mixture. We replicated each sample four times in all assays, with corresponding standards and controls, and averaged (median) the readings before statistical analysis. These fluorometric and colorimetric measurements represent rates of substrate consumption (nmol g^−1^ soil h^−1^), in response to underlying enzymatic reactions that are temperature sensitive (Saiya‐Cork et al. 2002; German, Weintraub, et al. 2011). So, we ensured that soil slurry and substrate were incubated at representative ambient temperatures at the study site for the respective months (i.e., 15°C for May; 25°C for July; 15°C for September).

### 2.3 Decomposer Biomass

We used three metrics of decomposer biomass, namely, microbial biomass carbon (MBC, mg C g^−1^ soil), microbial biomass nitrogen (MBN, mg N g^−1^ soil), and basal respiration (BR, mg C g^−1^ soil day^−1^). We used chloroform fumigation‐extraction to measure MBC and MBN (Jenkinson and Powlson 1976; Robertson et al. 1999; Bagchi et al. 2017). Briefly, we pre‐incubated 4 g soil for 24 h at 60% water holding capacity. After pre‐incubation, we fumigated the soils with ethanol‐free chloroform for 24 h in a desiccator under partial vacuum. After fumigation, we removed residual chloroform through aeration for 24 h. We then extracted the carbon and nitrogen from the soils with 0.05 M K_2_SO_4_, and then measured them in a TOC/TN analyzer (Shimadzu LCPH/CPN, Japan). MBC and MBN were calculated as the difference in C and N between fumigated samples and corresponding unfumigated controls. We considered an extraction efficiency of 0.45 for MBC and 0.54 for MBN (Beck et al. 1997). We measured BR with the alkali trap method for CO_2_ respired by soil microbes (Robertson et al. 1999; Bagchi et al. 2017). Briefly, we took 4 g of dry and sieved soil in a container and pre‐incubated at 60% water holding capacity for 24 h. Subsequently, we kept a beaker with 1 N KOH solution in the container and then sealed the container. We incubated the sealed container for 24 h at 28°C. After the incubation, we added 1 mL of 15% BaCl_2_ to the KOH. Finally, we titrated 2 mL of KOH solution with 0.1 N HCl in the presence of phenolphthalein indicator to calculate CO_2_ released from the soil with respect to corresponding controls.

### 2.4 Abiotic Soil Variables

We also measured eight key abiotic edaphic variables, namely, soil pH, electrical conductivity, bulk density, water holding capacity, and texture for soils in all the land‐use categories, following standard procedures (Robertson et al. 1999), in addition to recording the elevation (m) of the sampling locations.

### 2.5 Statistical Analysis

We investigated whether the differences between land‐use categories (H1–H4, above) were influenced by underlying spatial structure in the data. For this, we estimated the distance‐decay relationship for all the biotic variables. Next, we estimated the underlying spatial structure in the data using Moran's Eigenvector Maps (MEM) to analyze the role of spatial autocorrelation in determining the patterns in decomposer functions (Dray et al. 2006; Griffith and Peres‐Neto 2006; Legendre et al. 2015; Brind'Amour et al. 2018).

We used partial redundancy analysis (RDA) to estimate the relative importance of time (growth season), space (clustering), the eight abiotic variables (geo‐edaphic background), and land‐use category (human influence) in explaining variation among the biotic variables. A baseline RDA model contained the spatial and abiotic information from the sampling locations as explanatory variables. Spatial information on the latitude/longitude of the sampling locations was expressed in Universal Transverse Mercator (UTM) coordinates. The abiotic variables were soil pH, electrical conductivity, bulk density, water holding capacity, texture (sand, silt, and clay), and elevation. To this baseline model, we added time (3 months during the growth season) and land‐use type (three categories) to quantify the variance explained by them separately, as well as when they are included simultaneously.

To address the specific hypotheses (H1–H4), we first investigated the effect of seasonality (months, 3 levels) and land‐use (3 levels) on decomposer functions and multifunction using general linear models with repeated measures (GLM). The identity of each plot within its respective watershed was modeled as a random effect because measurements were repeated throughout the growing season. Here we checked whether the land‐use type and season explained variation in functions (*α* = 0.05), either as a main effect or through their 2‐way interaction. On the basis of observed and theoretical quantiles of residuals (Q–Q plots), data on decomposer functions and biomass required ln‐transformation to meet the assumption of homogeneity of residuals. From the seven individual decomposer functions, we estimated a multifunctionality index (Quero et al. 2013; Chandregowda et al. 2018). We calculated the standardized *z*‐score (overall mean of 0 and SD of 1) for each function in each land‐use for each month. Subsequently, we calculated the average of all the standardized (*z*‐score) ecosystem functions in each land‐use for each month and used that as a multifunctionality index. This helped equalize the variance among different functions and sampling points.

Next, we estimated a multivariate measure of functional diversity, using dispersion to estimate variation in decomposer functions in multivariate space (Anderson et al. 2006). We used ordination with principal coordinates analysis (PCoA) of pairwise Bray–Curtis dissimilarity to visualize decomposer functions across land‐use and seasons in a multivariate space summarized as two axes (Legendre and Legendre 2012). We calculated multivariate dispersion in the decomposer functions relative to their group centroids in multivariate space (Anderson et al. 2006) with the “betadisper” function of the “vegan” package in R.

We estimated stability of decomposer biomass—MBC, MBN, and BR—using indices of temporal invariance (Pimm 1984; Lehman and Tilman 2000; Wang and Loreau 2014). We calculated invariance as $\frac{\bar{x}}{s}$, where $\bar{x}$ is the mean biomass under a land‐use across the growth season and s is the standard deviation around the mean for a land‐use across the growth season.

We assessed the relationship between functional diversity and stability using structural equation models (SEM). SEMs help evaluate whether data support the presence of hypothesized causal relationships or not (Grace 2006). We built the model using paths from a priori examples in the literature. These paths evaluated whether changes in land‐use alter functional diversity and stability directly (Land‐use → Functional diversity → Stability) or indirectly through edaphic factors (Land‐use → Edaphic → Functional diversity → Stability). The edaphic variables were elevation, soil electrical conductivity, bulk density, pH, and texture (Figure S2). For soil texture, we only included sand and silt content in the analysis because of autocorrelation with clay content (Figures S3 and S4). All the paths were modeled as linear mixed‐effects using the “nlme” library. We performed SEM for native‐to‐livestock and for native‐to‐croplands changes separately. We assessed goodness of fit between the model and data with Fisher's C statistic. We implemented paths for SEM using the “piecewiseSEM” library in R (Lefcheck 2016).

All analyses were performed in R 3.6.3 using “nlme”, “vegan”, “ape”, and “spdep” packages.

## 3 Results

### 3.1 Seasonal Versus Spatial Variation (H1 and H1′)

For the abiotic variables, we did not detect any systematic variation among the three land‐use types in their soil pH, electrical conductivity, bulk density, water holding capacity, and texture (Figures S2 and S3). However, croplands had a narrower elevational range (4149 ± 36 m, mean ± SD) than the livestock (4574 ± 142 m) and native sites (4316 ± 293 m, Figure S2).

MEM revealed weak spatial structure among the biotic variables (Moran's coefficient between −0.93 and 1.04, *p* > 0.05 in most cases; Figures S5 and S6). This implies that two locations that are close to one another are not necessarily also equally similar in their biotic characteristics (Figures S5 and S6). The baseline RDA (Table 2) showed that variation in decomposer functions was neither explained by spatial autocorrelation (*F*_2,112_ = 0.89, *p* = 0.502) nor by background influence from the abiotic variables (*F*_7,107_ = 0.93, *p* = 0.516). The inclusion of growth season in the baseline RDA explained 41.2% of the variation (*F*_11,103_ = 6.56, *p* = 0.001). Including land‐use led to a moderate improvement in explanatory power in the full model with all variables (44.9% explained, *F*_13,101_ = 6.32, *p* = 0.001; Table 2, Figure S7).

**TABLE 2:** Summary of partial redundancy analysis (RDA).

RDA model	% Explained	*F*	*p*
Spatial	1.56	*F*_2,112_ = 0.89	0.502
Abiotic	5.73	*F*_7,107_ = 0.93	0.516
Spatial + Abiotic (Baseline Model)	7.09	*F*_9,105_ = 0.89	0.629
Spatial + Abiotic + Season	41.19	*F*_11,103_ = 6.56	0.001
Spatial + Abiotic + Land‐use	11.06	*F*_11,103_ = 1.17	0.236
Spatial + Abiotic + Season + Land‐use (Full Model)	44.86	*F*_13,101_ = 6.32	0.001

*Note:* Spatial information contains the latitude/longitude of the sampling locations expressed in Universal Transverse Mercator coordinates. The abiotic variables were soil pH, electrical conductivity, bulk density, water holding capacity, texture (sand, silt, and clay), and elevation. Growth season had three levels, that is, May, July, and September. Land‐use had three levels, that is, Native, Livestock, and Cropland.

### 3.2 Intensity of Decomposer Functions (H2 and H2′)

**FIGURE 2:**
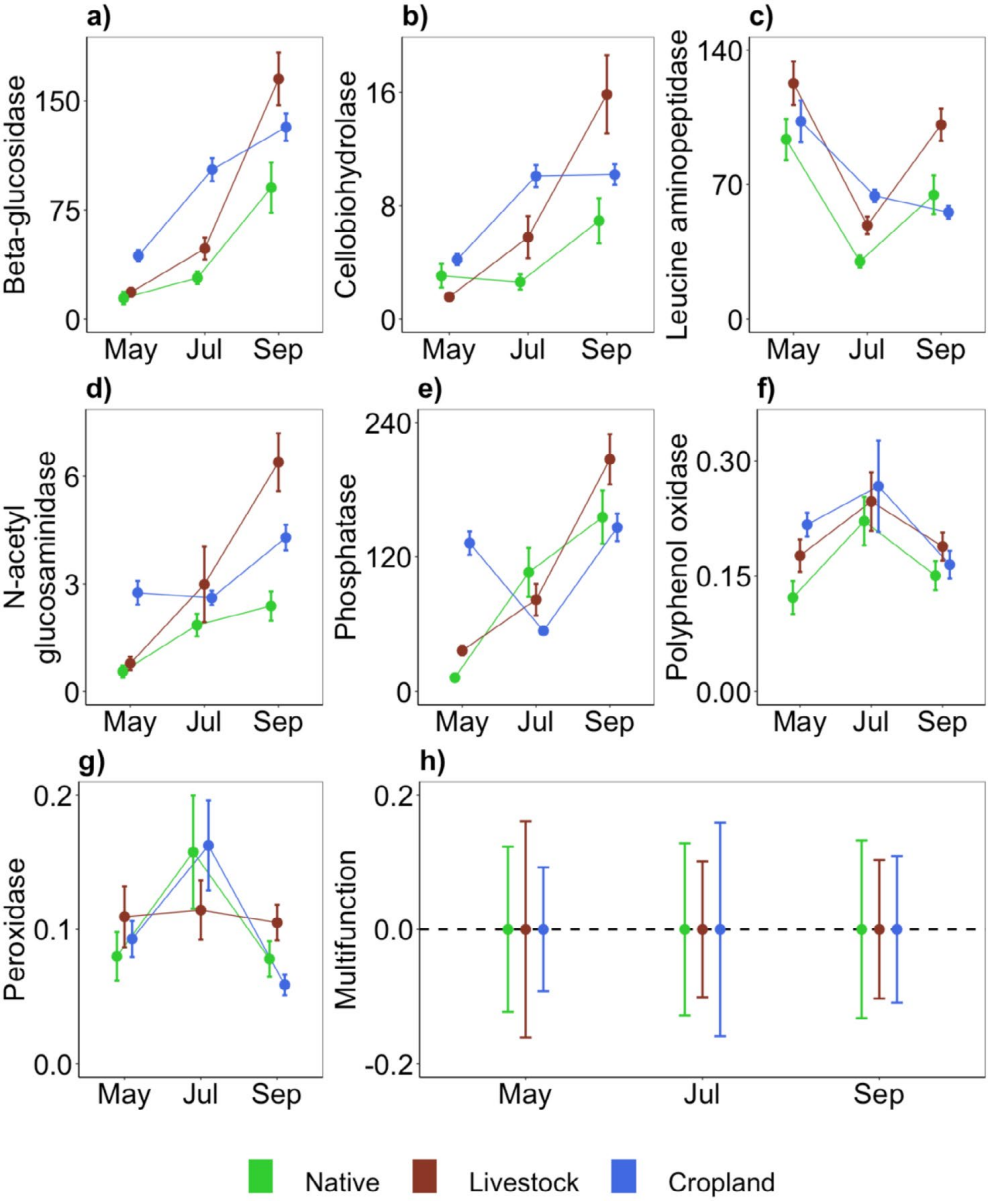
Seasonal profiles of seven decomposer functions (mean ± SE) across months in three land‐use types (a–g). Variation in aggregate estimate of multifunction (h). Many individual functions were higher for croplands, compared to native and livestock states. All decomposer functions show variation over the growth season. Multifunction did not vary with land‐use or by season. Beta‐glucosidase (a), Cellobiohydrolase (b), Leucine aminopeptidase (c), N‐acetylglucosaminidase (d), Phosphatase (e), Polyphenol oxidase (f), and Peroxidase (g) are expressed as nmol g^−1^ soil h^−1^. Multifunction (h) is unitless.

GLMs showed that growth season influenced all decomposer functions, either as a main effect or through a two‐way interaction with land‐use (Figure 2, Table S2). Two‐way interactions were found for BG (*F*_4,91_ = 8.41, *p* < 0.0001), CBH (*F*_4,90_ = 9.49, *p* < 0.0001), leucine aminopeptidase (*F*_4,91_ = 10.10, *p* < 0.0001), N‐acetylglucosaminidase (*F*_4,88_ = 10.51, *p* < 0.0001), and phosphatase (*F*_4,91_ = 42.60, *p* < 0.0001). The main effect of growth season was found on polyphenol oxidase (*F*_2,91_ = 5.34, *p* = 0.006) and on peroxidase (*F*_2,68_ = 3.97, *p* = 0.024). Influence of land‐use type was found on all dependent variables except polyphenol oxidase and peroxidase (Figure 2, Table S2). Seen together, the intensity of decomposer functions varied across the growth season, but the direction depends on land‐use type as native, livestock, or cropland relate to different responses in these functions. The intensity of decomposer functions was not necessarily negatively impacted by land‐use change. Multifunction index did not vary with land‐use and did not change with growth season (Figure 2, Table S2).

### 3.3 Heterogeneity of Decomposer Functions (H3 and H3′)

**FIGURE 3:**
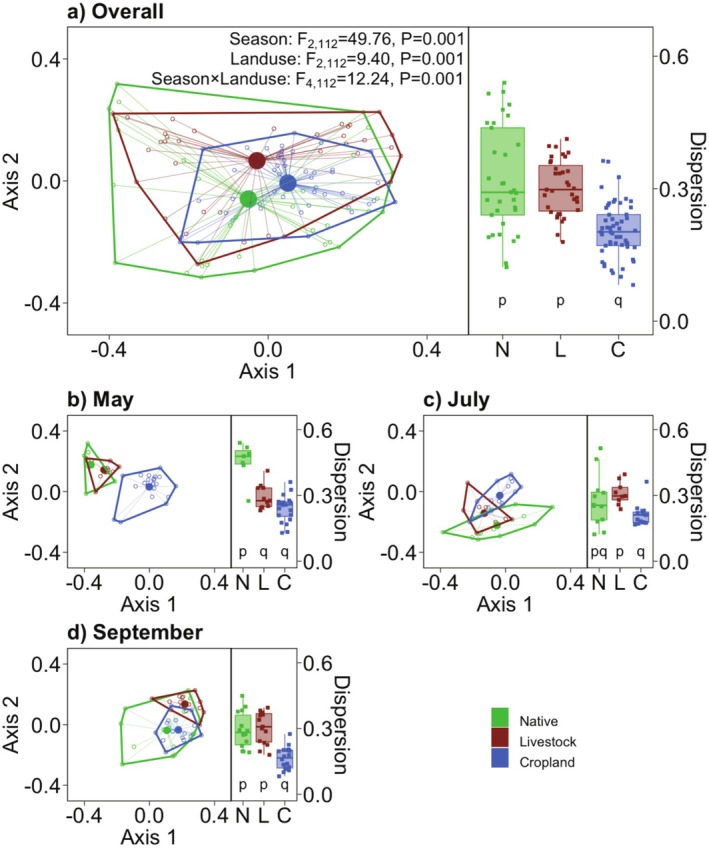
Heterogeneity in seven decomposer functions in multivariate space summarized as two PCoA axes. Croplands (C) are least heterogeneous, overall, as well as at each point in the growth season. Heterogeneity under livestock (L) is comparable to the reference native (N) state. Polygons denote the functional space occupied by each land‐use category, filled circles represent group centroids, and open circles are individual samples. Dispersion of individual samples from their respective group‐centroids as a measure of microbial functional diversity in multivariate space. Dispersion was lower for cropland compared to the native state—overall as well as in mid and late season. Dispersion under livestock is comparable to the reference native state. Lower‐case letters denote differences in pairwise‐comparisons (*α* = 0.05, Tukey's HSD).

The agroecosystems had lower heterogeneity of decomposer functions than the native state, as multivariate dispersion was low (Figure 3). In multivariate space, differences between group‐centroids were explained by a two‐way interaction between land‐use and season (PERMANOVA: *F*_4,112_ = 12.24, *p* = 0.001). Dispersion (diversity) of biotic functions was lower for cropland in comparison to the native and livestock state across the growth season (*F*_4,64_ = 6.43, *p* < 0.001, Figure 3). Overall, livestock sites were similar to the native state, whereas croplands were a subset of the native state (Figure 3).

### 3.4 Relationship Between Functional Diversity and Stability (H4 and H4′)

**FIGURE 4:**
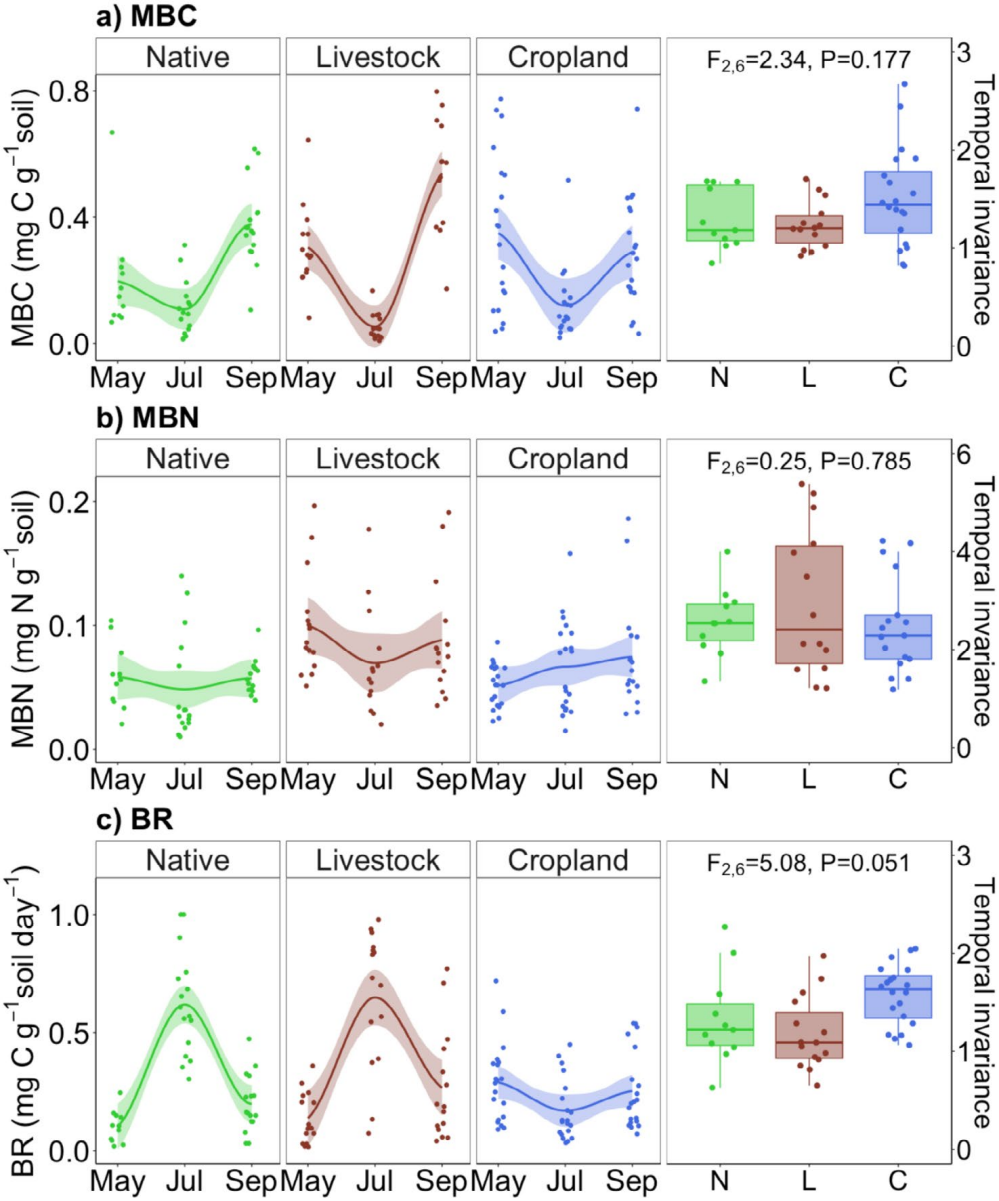
Seasonal variation through the growth season in three measures of decomposer biomass: Microbial biomass carbon (MBC, a), microbial biomass nitrogen (MBN, b), and basal respiration (BR, c) across different land‐use types that are native (N), livestock (L), and cropland (C). There was high variation through the growth season in decomposer biomass, in each land‐use type. Trendline for each case is overlaid (loess smoothing, ±1 SE). Stability of decomposer biomass through the growth season, estimated as temporal invariance in MBC (a), MBN (b), and BR (c). Stability under livestock and croplands did not differ from the reference native state.

All three measures of decomposer biomass (MBC, MBN, and BR) varied substantially through the growth season and between land‐use types (Figure 4). There was a significant two‐way interaction between land‐use and growth season in MBC (*F*_4,90_ = 7.01, *p* = 0.0001) and BR (*F*_4,91_ = 17.06, *p* < 0.0001). MBN varied with growth season (*F*_2,87_ = 3.95, *p* = 0.022), but not by land‐use (*F*_2,7_ = 4.69, *p* = 0.051). Land‐use did not alter the temporal invariance of decomposer biomass (Figure 4). All three measures of the temporal stability of biomass through the growth season did not differ by land‐use type (*p* > 0.05 in each case, Figure 4).

**FIGURE 5:**
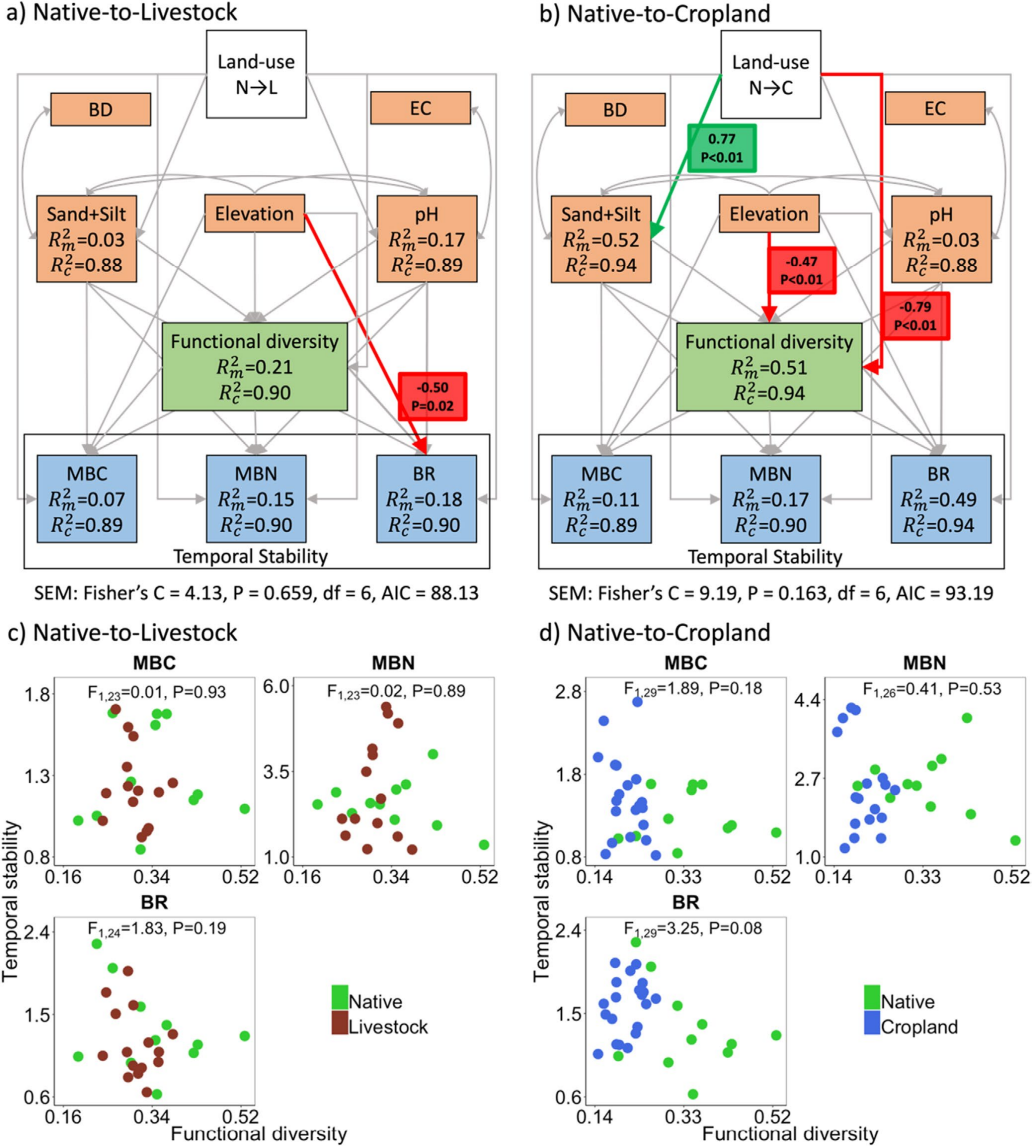
Path analysis using mixed‐effects structural equation model to evaluate the relationship between land‐use change, edaphic factors, functional diversity, and stability (a) when native herbivores (N) are replaced by livestock (L) and (b) when native grazing lands are repurposed to grow crops (C). Unidirectional arrows represent hypothesized causal paths, and bidirectional arrows indicate correlated paths. Thick arrows (green and red) are statistically significant paths; thin gray arrows are paths that were statistically non‐significant (*α* = 0.05). Standardized path coefficients and their statistical significance are shown alongside marginal and conditional *R* ^2^ for different variables. BD, bulk density; BR, Basal respiration; EC, electrical conductivity; MBC, microbial biomass carbon; MBN, microbial biomass nitrogen. Pair‐wise relationship between three measures of stability and microbial functional diversity when L replaces N (c) and when C replaces N (d). Stability is decoupled from the functional diversity of microbial decomposers.

SEMs (Figure 5) indicated a good match between the data and hypothesized paths for both livestock (Fisher's *C* = 4.13, *p* = 0.66, df = 6) and cropland agroecosystems (Fisher's *C* = 9.19, *p* = 0.16, df = 6). Land‐use change from native (N) to cropland (C) coincided with reduced functional diversity (Land‐use (N to C) → Functional diversity), but native to livestock (L) change did not. Neither land‐use change altered the stability of decomposer biomass either directly or indirectly through edaphic factors and functional diversity. Additionally, we find that soil texture variability, with greater sand and silt content, also plays a role (Land‐use (N to C) → Soil texture). We also find that in the native to livestock SEM, elevation decreases the stability of decomposer biomass through BR (Elevation (N to L) → BR). In the native to cropland SEM, elevation decreases functional diversity alongside the direct negative effect of land‐use change (Elevation (N to C) → Functional diversity).

## 4 Discussion

Generally, the loss of functions attributed to land‐use change compromises the stability of ecosystem services related to primary and secondary production (Cariveau et al. 2013; Blüthgen et al. 2016; Olivier et al. 2020; Zhou et al. 2020). We attempt to determine whether an equivalent generalization can also apply to decomposer functions. Since decomposition processes are remarkably similar in both natural and agroecosystems, this can have implications for the stewardship of agroecosystems (Tscharntke et al. 2005; Cardinale et al. 2012; Wood et al. 2015). We find that only some, and not all, knowledge gained for functional diversity in producers and consumers can be extended to decomposers. As expected, land‐use such as simplified agroecosystems indeed show homogenized decomposer functions (Figures 2 and 3). However, this does not necessarily have negative impacts on individual functions and multifunction, as these are maintained at levels comparable to or exceeding the reference natural state (Figure 2). Additionally, the stability of decomposer functions is not compromised in agroecosystems (Figure 4). In this way, the response of decomposer functions to human land‐use appears fundamentally different from what is found in producers and consumers (Zhou et al. 2020). First, we find support for H1, and not H1′ (Figure 2), where decomposer functions are indeed more variable through season than across space, consistent with previous studies (Bardgett et al. 2005; Frossard et al. 2012; Roy, Naidu, et al. 2023; Roy, Karapurkar, et al. 2023). Second, we find support for H2′ instead of H2 as decomposer functions and multifunction were not lower in the agroecosystems compared to the reference native state (Figure 2). Third, we find support for H3, and not H3′, where agroecosystems appear homogenous compared to the native state (Figure 3). Fourth, we find support for H4′ instead of H4 as functional diversity in decomposers appears to be decoupled from the stability of decomposer biomass across seasons (Figures 4 and 5).

The effect of land‐use change on decomposer functions should be assessed independent of any underlying spatial structure in the data. Under strong spatial structuring, samples from nearby locations will be more similar than from distant ones (Legendre 1993; Sokal et al. 1998). This could occur due to autocorrelation that arises from pair‐wise differences in geographic distance, and non‐independence from coupled variation in the background abiotic environment (Legendre et al. 2002). We find that variation in decomposer functions is attributable to land‐use and growth season, but not to background spatial autocorrelation or abiotic edaphic conditions (Figures S2–S7, Table 2). Overall, the lack of any overwhelming influence of spatial structure allows inference on how decomposer functions respond to land‐use (Figures 2, 3, 4, 5).

Our results show that land‐use change, particularly croplands, reduces the variability in microbial functions (Figure 3), and results in functional homogenization (Olden et al. 2004; Clavel et al. 2011). Functional homogenization is perceived as the loss of “specialist” species with a gain in “generalists” (Olden et al. 2004; Clavel et al. 2011). Nutrient enrichment due to changes in land‐use is known to favor microbial communities with generalist copiotrophs over specialist oligotrophs (Leff et al. 2015; Ho et al. 2017). At our study site, land‐use change alters the quality of biomass input to the soil. Croplands receive manure that changes the nutrient status of soils compared to both the reference and pastoral states. Thus, the replacement of specialist (oligotrophs) microbes by generalists (copiotrophs) because of nutrient enrichment may lead to the homogenization of decomposer functions (Olden et al. 2004; Clavel et al. 2011), but the underlying mechanisms are yet to be fully understood.

Our results for decomposers are inconsistent with some familiar patterns observed in producers and consumers (Figures 4 and 5). Changing the identity of consumers to create the pastoral state, where native herbivores are replaced by livestock, did not lead to substantial differences in functions and their functional diversity, as well as stability (Griffiths et al. 2008; De Vries et al. 2012; Griffiths and Philippot 2013; Briske 2017). Previous studies have encountered similar ecological stability in grasslands when the stability of producers was not influenced by consumers (Ren et al. 2018; Valone and Balaban‐Feld 2019; Kohli et al. 2019). We find that ecological stability manifests in decomposers as well. On the other hand, more drastic alterations to create monocultures of crops do decrease functional diversity, but without negative impacts on stability. This contrasts with the patterns observed for producers, where other types of human impacts weaken diversity‐stability relationships (Hautier et al. 2014; Bharath et al. 2020). Another noteworthy departure from the familiar patterns is that decomposer functions tended to be higher relative to the native state, and this is a stark difference between the aboveground and belowground realms. Such decoupling between functional diversity and stability can be ubiquitous in hyper‐diverse communities where different organisms can perform a common set of functions. As this may impart functional redundancy, small to moderate changes in taxonomic diversity may not impact functional diversity (Roy, Karapurkar, et al. 2023). However, it remains to be seen whether the disconnect between microbial functional diversity and ecosystem stability could be recapitulated in agroecosystems that are more intensively managed, for instance, farming with chemical fertilizers and livestock production systems with heavy usage of veterinary drugs.

Decomposition processes showed remarkable resistance to land‐use change, particularly under livestock, where the intensity, heterogeneity, and stability remained comparable to the native state. Such ecological resistance could be one reason why traditional pastoralist systems have survived on all continents for millennia, whereas more intensive livestock‐production systems (e.g., modern ranching operations) are more susceptible to environmental fluctuations. This contrast becomes even more relevant in the light of future climate projections (Briske 2017; Derner et al. 2018). Rising demands for livestock products due to evolving human diets, socio‐economic growth, and globalization (Godfray et al. 2010; Poore and Nemecek 2018) posit that the stability and resistance of livestock‐production systems should become assets as well as targets for management and stewardship (Briske 2017). The ability to meet future demands from such agroecosystems hinges upon how decomposer functions, multifunction, functional diversity, and stability are translated into services. In this way, decomposer functions can support the stewardship of agroecosystems using the principles developed in natural systems (Tscharntke et al. 2005; Cardinale et al. 2012; Wood et al. 2015). Thus, the decomposer connection in the relationship between functional diversity and stability could be incorporated into sustainability policies for agroecosystems, especially in changing environments.

## 5 Conclusion

Our study shows that the positive linkages between functional diversity and ecosystem stability learned from producers and consumers in natural ecosystems cannot be extended to microbial decomposers in agroecosystems. We find that the ecosystem stability is decoupled from the functional diversity of decomposers when natural ecosystems are repurposed into two types of agroecosystems (livestock grazing and croplands). Our results demonstrate ecological resistance to land‐use change from natural to agroecosystems. Despite changes in functional diversity, land‐use change did not alter ecosystem stability. Further, we find that land‐use change did not reduce the intensity of decomposer functions, which suggests ecological resistance. These findings highlight the importance of microbial decomposers toward stewardship in agroecosystems.

## Author Contributions

**Shamik Roy:** conceptualization (equal), formal analysis (lead), investigation (lead), methodology (equal), visualization (lead), writing – original draft (equal), writing – review and editing (equal). **Sumanta Bagchi:** conceptualization (equal), funding acquisition (lead), investigation (supporting), methodology (supporting), supervision (lead), writing – original draft (equal), writing – review and editing (equal).

## Conflicts of Interest

The authors declare no conflicts of interest.

## Supporting information


**Figure S1:** Description of strong seasonality in the Spiti region of northern India in terms of precipitation (a), temperature (b), and plant biomass as satellite‐derived normalized difference vegetation index, NDVI (c), between 2005 and 2018. Red points represent the values for 2017.
**Figure S2:** Different abiotic variables across three land‐use categories—native, livestock, and croplands, near village Kibber in Spiti region of northern India.
**Figure S3:** Soil texture across three land‐use categories—native, livestock, and croplands, near village Kibber in Spiti region of northern India. All the land‐use categories have coarse soil texture (sandy‐loam), as sand content is usually > 65% and clay content < 18%.
**Figure S4:** Correlation plot between abiotic variables. The abiotic variables were soil pH, electrical conductivity (EC), bulk density (BD), water holding capacity (WHC), texture (sand, silt, and clay), and elevation.
**Figure S5:** Distance–decay relationships between different biotic and abiotic response variables (Euclidean Distance) and geographical separation.
**Figure S6:** Spatial structure depicted as Moran Eigenvector Map (MEM) for different decomposer functions across seasons for three land‐use categories. Dotted vertical lines depict the bounds of the spatial weighting matrix computed for our sampling locations. Black bars represent Moran's coefficient is statistically significant (*p* < 0.05), whereas white bars represent it is not significant (*p* > 0.05). Overall, spatial structure in decomposer functions is generally weak.
**Figure S7:** Variance partitioning as a Venn diagram among three groups of explanatory variables—abiotic, growth season, and land‐use—that account for variation in the biotic variables. The abiotic variables were soil pH, electrical conductivity, bulk density, water holding capacity, texture (sand, silt, and clay), and elevation. Growth season had three levels, that is, May, July, and September. Land‐use had three levels, that is, Native, Livestock, and Cropland.
**Table S1:** Summary description of seven extracellular enzymes that perform decomposer functions in soil, their International Union of Biochemistry and Molecular Biology (IUBMB) classification code, and colorimetric/fluorogenic substrate used for estimating their activity.
**Table S2:** Summary of GLM for individual decomposer functions, multifunction, measures of functional heterogeneity, and stability of decomposer biomass.

**Data S1:** ece372190‐sup‐0002‐DataS1.csv.

## Data Availability

All the required data are uploaded as Supporting Information.
