# Supplementary material for: Decoupling Between Functional Diversity and Stability of Decomposer Functions in Natural and Agroecosystems Can Favor Resistance to Land‐Use Change
Source: Ecol Evol. 2025 Sep 20;15(9):e72190. doi: 10.1002/ece3.72190 (PMC12449671; doi:10.1002/ece3.72190)
Supplement: Supplementary file 1 — Figure S1: Description of strong seasonality in the Spiti region of northern India in terms of precipitation (a), temperature (b), and plant biomass as satellite‐derived normalized difference vegetation index, NDVI (c), between 2005 and 2018. Red points represent the values for 2017. Figure S2: Different abiotic variables across three land‐use categories—native, livestock, and croplands, near village Kibber in Spiti region of northern India. Figure S3: Soil texture across three land‐use categories—native, livestock, and croplands, near village Kibber in Spiti region of northern India. All the land‐use categories have coarse soil texture (sandy‐loam), as sand content is usually > 65% and clay content < 18%. Figure S4: Correlation plot between abiotic variables. The abiotic variables were soil pH, electrical conductivity (EC), bulk density (BD), water holding capacity (WHC), texture (sand, silt, and clay), and elevation. Figure S5: Distance–decay relationships between different biotic and abiotic response variables (Euclidean Distance) and geographical separation. Figure S6: Spatial structure depicted as Moran Eigenvector Map (MEM) for different decomposer functions across seasons for three land‐use categories. Dotted vertical lines depict the bounds of the spatial weighting matrix computed for our sampling locations. Black bars represent Moran's coefficient is statistically significant (p < 0.05), whereas white bars represent it is not significant (p > 0.05). Overall, spatial structure in decomposer functions is generally weak. Figure S7: Variance partitioning as a Venn diagram among three groups of explanatory variables—abiotic, growth season, and land‐use—that account for variation in the biotic variables. The abiotic variables were soil pH, electrical conductivity, bulk density, water holding capacity, texture (sand, silt, and clay), and elevation. Growth season had three levels, that is, May, July, and September. Land‐use had three levels, that is, Native, Livest [file ECE3-15-e72190-s001.docx]

SUPPLEMENTARY MATERIAL

**Decoupling between functional diversity and stability of decomposer functions in natural and agroecosystems can favour resistance to land-use change**

CONTENTS

Fig. S.1. Summary of climatic variables

Fig. S.2. Summary of abiotic variables

Fig. S.3. Summary of soil texture

Fig. S.4. Correlation between abiotic variables

Fig. S.5. Relationship between response variables and geographical distance

Fig. S.6. Summary of Moran Eigenvector Map (MEM) for decomposer functions

Fig. S.7. Variance partitioning

Table S.1. Summary description of seven extracellular enzymes

Table S.2. Summary of GLM.

Fig. S.1. Description of strong seasonality in Spiti region of northern India in terms of precipitation (**a**), temperature (**b**), and plant biomass as satellite-derived normalized difference vegetation index, NDVI (**c**), between 2005-2018. Red points represent the values for 2017.


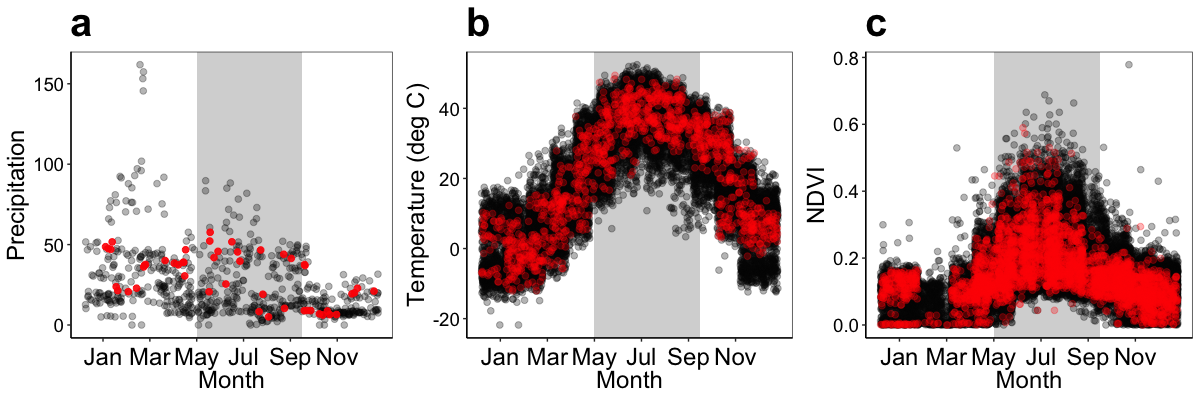


Fig. S.2. Different abiotic variables across three land-use categories – native, livestock, and croplands, near village Kibber in Spiti region of northern India.


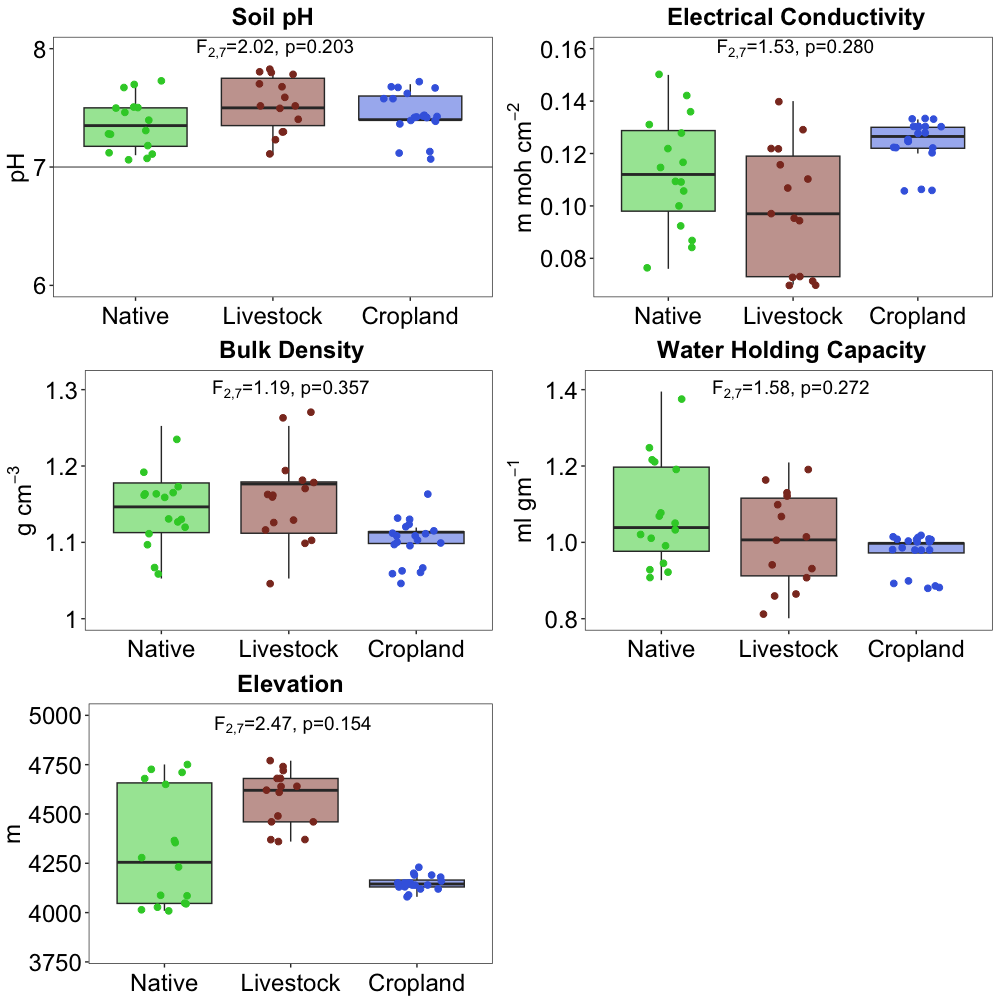


Fig. S.3. Soil texture across three land-use categories – native, livestock, and croplands, near village Kibber in Spiti region of northern India. All the land-use categories have coarse soil texture (sandy-loam), as sand content is usually >65% and clay content <18%.


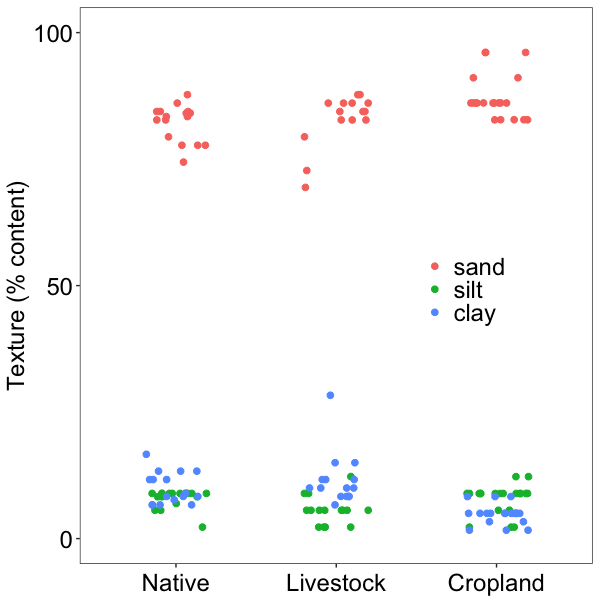


Fig S.4. Correlation plot between abiotic variables. The abiotic variables were soil pH, electrical conductivity (EC), bulk density (BD), water holding capacity (WHC), texture (sand, silt, and clay), and elevation.


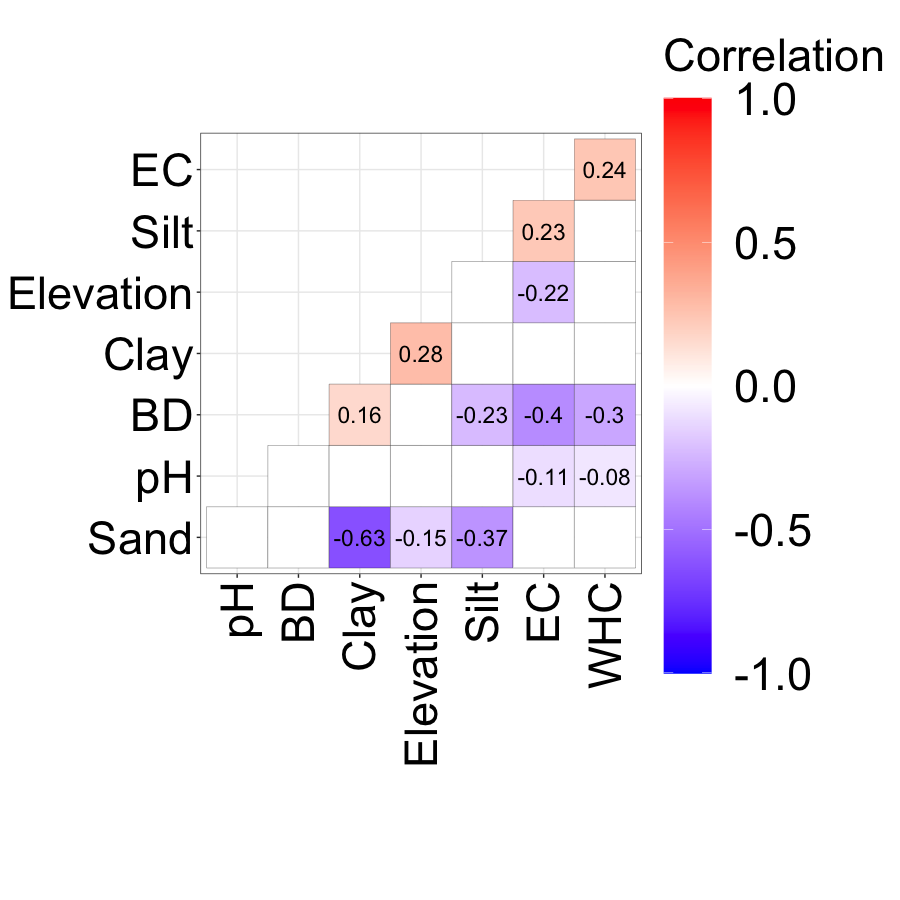


Fig. S.5. Distance-decay relationships between different biotic and abiotic response variables (Euclidean Distance) and geographical separation.


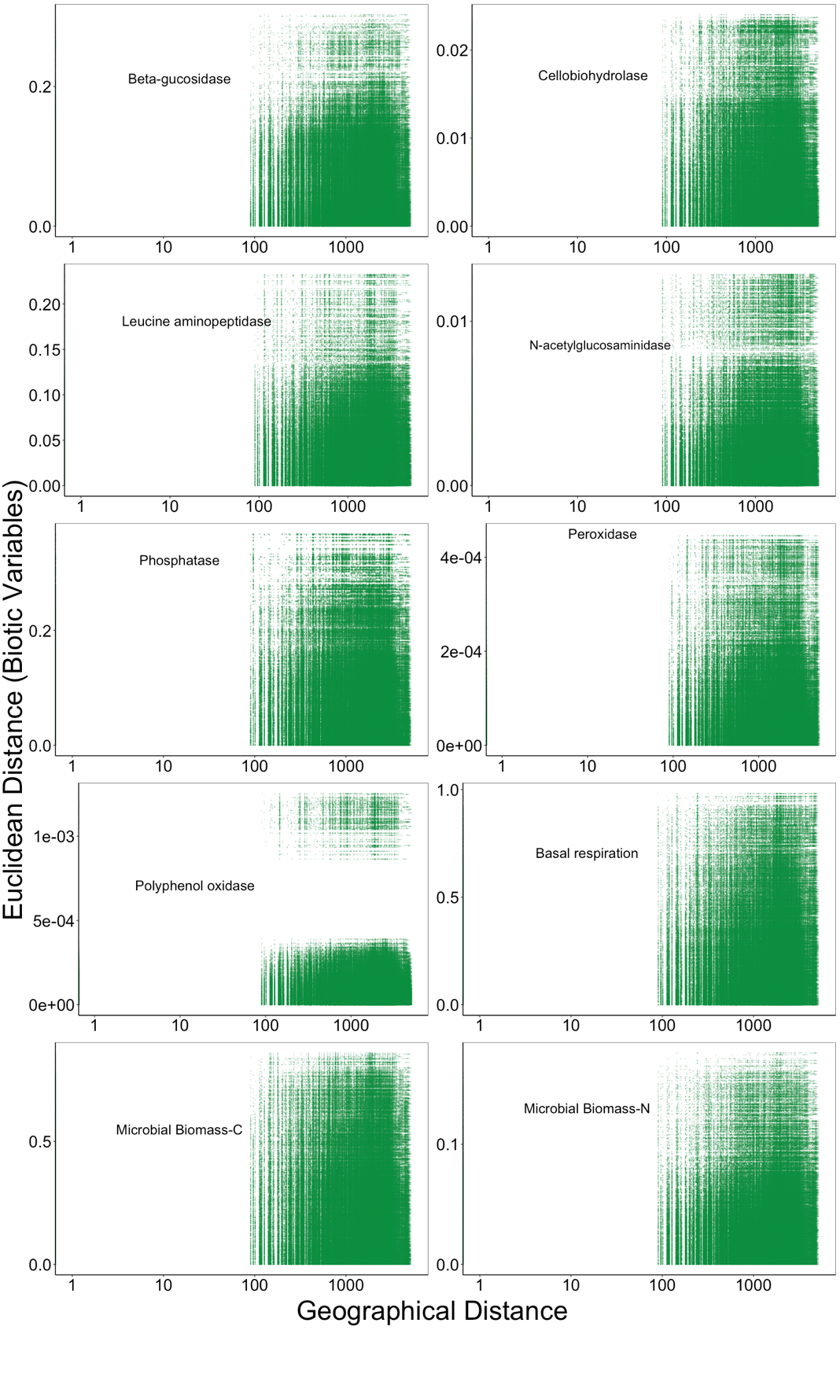


Fig S.6. Spatial structure depicted as Moran Eigenvector Map (MEM) for different decomposer functions across seasons for three land-use categories. Dotted vertical lines depict the bounds of spatial weighting matrix computed for our sampling locations. Black bars represent Moran’s coefficient is statistically significant (*P*<0.05), while white bars represent it is not significant (*P*>0.05). Overall, spatial structure in decomposer functions is generally weak. Abbreviations – BG: Beta-glucosidase, CBH: Cellobiohydrolase, LP: Leucine aminopeptidases, NAG: Beta-*N*-acetylglucosaminidase, P: Phosphatase, PO: Peroxidase, PPO: Polyphenol oxidase, BR: Basal respiration, MBC: Microbial Biomass-Carbon, and MBN: Microbial Biomass-Nitrogen.


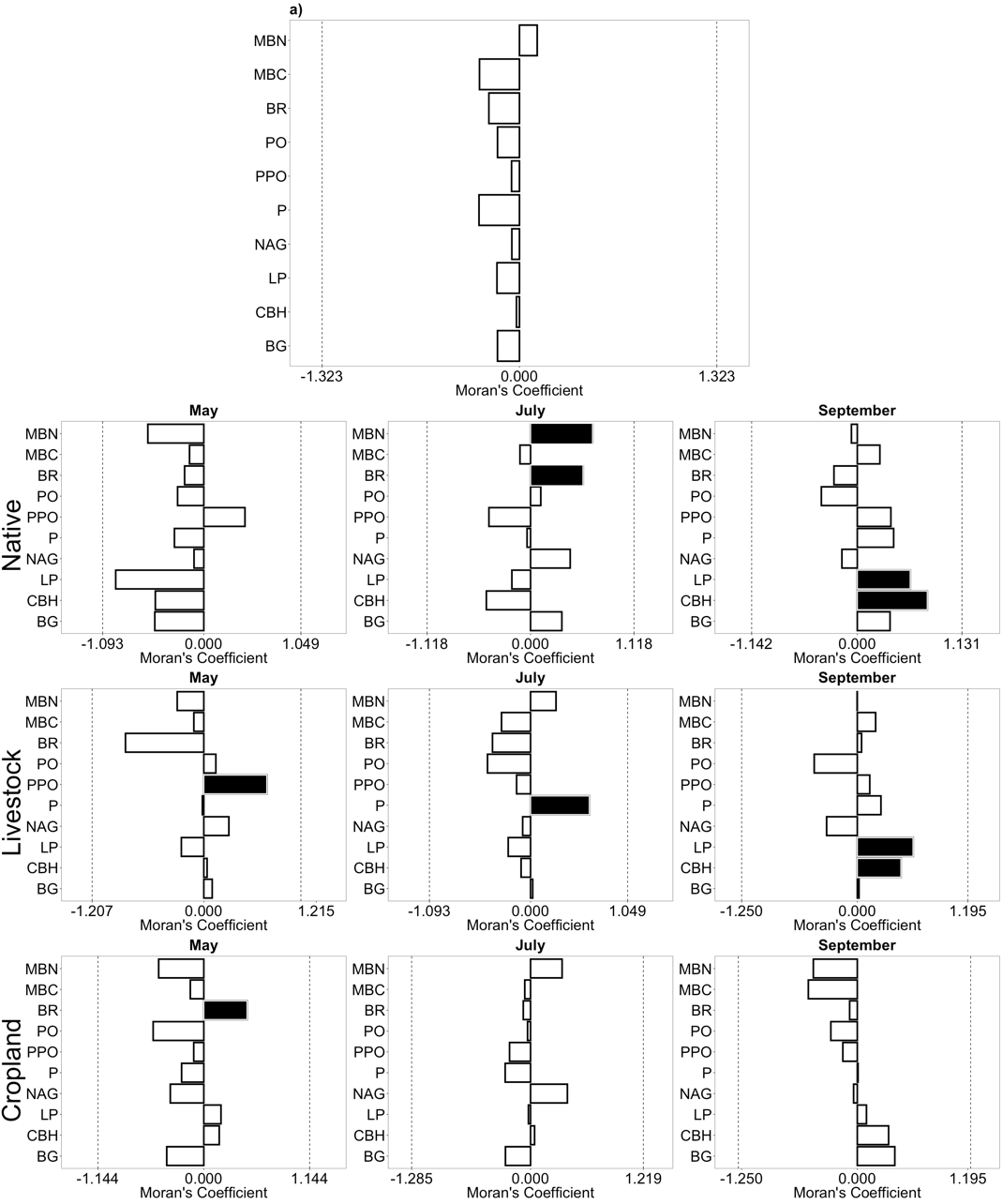


Fig S.7. Variance partitioning as a Venn diagram among three groups of explanatory variables - abiotic, growth-season, and land-use - that account for variation in the biotic variables. The abiotic variables were soil pH, electrical conductivity, bulk density, water holding capacity, texture (sand, silt, and clay), and elevation. Growth-season had three levels, i.e., May, July, and September. Land-use had three levels, i.e., Native, Livestock, and Cropland.


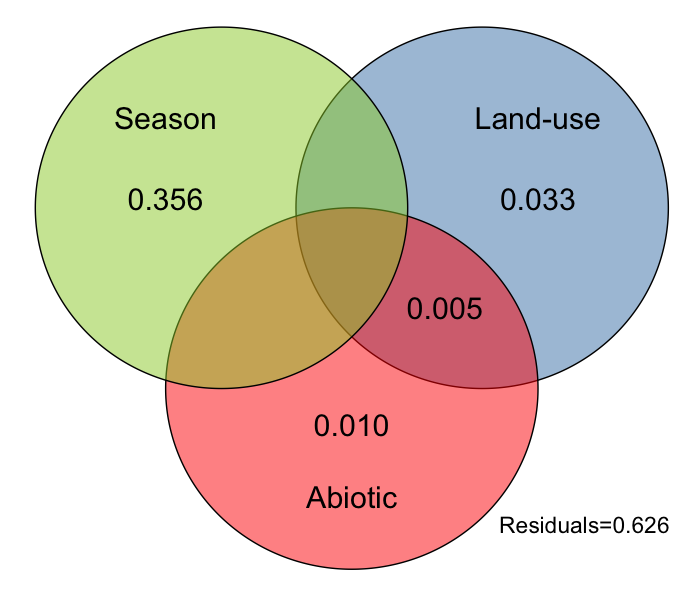


Table S.1. Summary description of seven extracellular enzymes that perform decomposer functions in soil, their International Union of Biochemistry and Molecular Biology (IUBMB) classification code, and colorimetric/fluorogenic substrate used for estimating their activity.

| **Extracellular enzyme** | **Target** | **IUBMB Class** | **Substrate** |
| --- | --- | --- | --- |
| Beta-Glucosidase (BG) | Carbon | 3.2.1.21 | 4-MUB β-D- glucopyranoside |
| Cellobiohydrolase (CBH) | Carbon | 3.2.1.91 | 4-MUB β-D-cellobioside |
| Leucine Aminopeptidase (LP) | Nitrogen | 3.4.11.1 | L-Leucine-7-amido-4-methylcoumarin hydrochloride |
| Beta-*N*-acetylglucosaminidase (NAG) | Nitrogen | 3.2.1.52 | 4-MUB N-acetyl-β-D-glucosaminide |
| Phophatase (P) | Phosphorous | 3.1.3.1 | 4-MUB phosphate |
| Polyphenol Oxidase (PPO) | Multipurpose | 1.10.3.1  1.10.3.2  1.13.11.1  1.13.11.2  1.14.18.1 | L-dihydroxyphenylalanine (DOPA) |
| Peroxidase (PO) | Recalcitrant organic matter | 1.11.1.7  1.11.1.13  1.11.1.14 | L-dihydroxyphenylalanine (DOPA)  Hydrogen peroxide |

Table S.2. Summary of GLM for individual decomposer functions, multifunction, measures of functional heterogeneity, and stability of decomposer biomass.

| **Variable** | **Effect** | **F-value** | **P-value** |
| --- | --- | --- | --- |
| Beta-glucosidase (Fig. 2a) | Season | F_2,91_=126.87 | <0.0001 |
|  | Land-use | F_2,7_=15.40 | 0.003 |
|  | Interaction | F_4,91_=8.41 | <0.0001 |
|  |  |  |  |
| Cellobiohydrolase (Fig. 2b) | Season | F_2,90_=44.57 | <0.0001 |
|  | Land-use | F_2,7_=28.85 | 0.0004 |
|  | Interaction | F_4,90_=9.49 | <0.0001 |
|  |  |  |  |
| Leucine aminopeptidase (Fig. 2c) | Season | F_2,91_=60.44 | <0.0001 |
|  | Land-use | F_2,7_=5.56 | 0.036 |
|  | Interaction | F_4,91_=10.10 | <0.0001 |
|  |  |  |  |
| N-acetylglucosaminidase (Fig. 2d) | Season | F_2,88_=48.22 | <0.0001 |
|  | Land-use | F_2,7_=29.33 | 0.0004 |
|  | Interaction | F_4,88_=10.51 | <0.0001 |
|  |  |  |  |
| Phosphatase (Fig. 2e) | Season | F_2,91_=80.79 | <0.0001 |
|  | Land-use | F_2,7_=2.36 | 0.164 |
|  | Interaction | F_4,91_=42.61 | <0.0001 |
|  |  |  |  |
| Polyphenol oxidase (Fig. 2f) | Season | F_2,91_=5.34 | 0.006 |
|  | Land-use | F_2,7_=4.11 | 0.066 |
|  | Interaction | F_4,91_=2.13 | 0.083 |
|  |  |  |  |
| Peroxidase (Fig. 2g) | Season | F_2,68_=3.97 | 0.024 |
|  | Land-use | F_2,7_=1.11 | 0.380 |
|  | Interaction | F_4,68_=1.41 | 0.238 |
|  |  |  |  |
| Multifunction (Fig. 2h) | Season | F_2,96_<0.01 | 1.000 |
|  | Land-use | F_2,7_<0.01 | 0.996 |
|  | Interaction | F_4,96_<0.01 | 1.000 |
|  |  |  |  |
| Microbial biomass-C (Fig. 4a) | Season | F_2,90_=61.38 | <0.0001 |
|  | Land-use | F_2,7_=0.13 | 0.879 |
|  | Interaction | F_4,90_=7.01 | 0.0001 |
|  |  |  |  |
| Microbial biomass-N (Fig. 4b) | Season | F_2,87_=3.95 | 0.022 |
|  | Land-use | F_2,7_=4.69 | 0.051 |
|  | Interaction | F_4,87_=2.12 | 0.084 |
|  |  |  |  |
| Basal respiration (Fig. 4c) | Season | F_2,91_=15.29 | <0.0001 |
|  | Land-use | F_2,7_=0.34 | 0.724 |
|  | Interaction | F_4,91_=17.06 | <0.0001 |
